# Recurrence of perinatal death in Northern Tanzania: a registry based cohort study

**DOI:** 10.1186/1471-2393-13-166

**Published:** 2013-08-29

**Authors:** Michael J Mahande, Anne K Daltveit, Blandina T Mmbaga, Joseph Obure, Gileard Masenga, Rachel Manongi, Rolv T Lie

**Affiliations:** 1 Kilimanjaro Christian Medical University College, Moshi, Tanzania; 2 Department of Global Public Health and Primary Care, University of Bergen, Bergen, Norway; 3 Centre for International Health, University of Bergen, Bergen, Norway; 4 Norwegian Institute of Public Health, Bergen, Norway; 5 Department of Obstetrics and Gynaecology, Kilimanjaro Christian Medical Centre, Moshi, Tanzania

**Keywords:** Perinatal mortality, Perinatal death, Recurrence, Risk factors, Continuation rate

## Abstract

**Background:**

Perinatal mortality is known to be high in Sub-Saharan Africa. Some women may carry a particularly high risk which would be reflected in a high recurrence risk. We aim to estimate the recurrence risk of perinatal death using data from a hospital in Northern Tanzania.

**Methods:**

We constructed a cohort study using data from the hospital based KCMC Medical Birth Registry. Women who delivered a singleton for the first time at the hospital between 2000 and 2008 were followed in the registry for subsequent deliveries up to 2010 and 3,909 women were identified with at least one more delivery within the follow-up period. Recurrence risk of perinatal death was estimated in multivariate models analysis while adjusting for confounders and accounting for correlation between births from the same mother.

**Results:**

The recurrence risk of perinatal death for women who had lost a previous baby was 9.1%. This amounted to a relative risk of 3.2 (95% CI: 2.2 - 4.7) compared to the much lower risk of 2.8% for women who had had a surviving baby. Recurrence contributed 21.2% (31/146) of perinatal deaths in subsequent pregnancies. Preeclampsia, placental abruption, placenta previa, induced labor, preterm delivery and low birth weight in a previous delivery with a surviving baby were also associated with increased perinatal mortality in the next pregnancy.

**Conclusions:**

Some women in Tanzanian who suffer a perinatal loss in one pregnancy are at a particularly high risk of also losing the baby of a subsequent pregnancy. Strategies of perinatal death prevention that target pregnant women who are particularly vulnerable or already have experienced a perinatal loss should be considered in future research.

## Background

Pregnancy complications are among of the major health problems in the developing world. Perinatal mortality is considered an important indicator of mother and child health care [1]. The World Health Organization (WHO) has estimated that around 5 million perinatal deaths occur each year, the majority of these in developing countries [2]. Recent community-based studies in Burkina-Faso and Uganda have reported consistently high perinatal mortality (79 per 1000 and 41 per 1000 births, respectively) [3,4]. In Tanzania, one recent hospital-based study estimated a perinatal mortality rate of 38 per 1000 births [5]. In a hospital-based study using data from Northern Tanzania, we found a perinatal mortality rate of 58 per 1000 live births among local non-referred births [6]. This is about ten-fold the perinatal mortality reported in western countries. In Norway, for example, the official perinatal mortality was 4.7 per 1000 births in the period from 2000 to 2010 (reported by the Medical Birth Registry of Norway [7].

Strategies to reduce this gap clearly need to address the overall resource situation and general health system characteristics. However, given the limited resources in African countries, it may be important to target groups of particularly vulnerable mothers and babies in attempts to reduce the mortality. One important tool to discover heterogeneity of risk and identify high risk individuals is identification of mothers who may experience recurrent losses [8]. There are, however, few studies on recurrence of perinatal deaths from African countries, and data with prospective follow up of mothers are lacking.

Prospective studies in western countries have reported two- to five-fold risks of perinatal death in subsequent pregnancies after a perinatal loss [9-11]. Against a low population risk in these countries, the absolute recurrence risk is still low. If similar levels of relative risk of recurrence exist in Africa against the much higher population risk, some women could carry an exceptionally high risk and might benefit from special prevention strategies. If, however, recurrence risk is not much higher than the average risk, strategies addressing the whole population may still be more important. The aim of the current paper is to investigate the recurrence risk of perinatal death using data from a hospital-based registry in Northern Tanzania.

## Methods

### Study design and sources of data

We designed a cohort study using existing but prospectively collected birth registry data from Kilimanjaro Christian Medical Centre (KCMC). This hospital is located in the Moshi urban district, Kilimanjaro region in the Northern Tanzania and is one of four zonal referral hospitals in Tanzania. It receives deliveries from the local community as well as referred cases from distant areas. The medical birth registry at KCMC was established as a pilot project in 1999 in collaboration with The Medical Birth Registry of Norway and the University of Bergen and it has been in regular operation since July 2000. Since then, data were prospectively collected on routine daily basis for all women who deliver at KCMC from 2000 to 2010.

The registry records information for all mothers who deliver in the obstetrics and gynecology department at KCMC within 24 hours after delivery or as soon as mothers have recovered in case of complicated deliveries. Data from the medical records were abstracted and trained midwife nurses conducted interviews on a routine daily basis for every woman who delivered in the hospital using a standardized form on paper. In addition, admitted mothers were asked to provide their antenatal care cards. Verbal consent was sought prior to the interview. All data were entered into a database system specially designed for the birth registry. The data entered in the registry include: Basic information concerning parents (socio-demographic characteristics), maternal health before and during present pregnancy, complications during labour and delivery, and information from the interview regarding the mother’s previous pregnancies. In addition, information on the baby such as sex, date and time of delivery, birth weight, gestational age, presentation, length and head circumference, plurality, mode of delivery, abnormal conditions (birth defects, injuries or other diseases), Apgar score, and child status in four categories: 1) live born 2) live born transferred to NCU 3) neonatal death in labour ward, 4) stillborn were recorded.

### Follow-up by record linkage

All women who deliver at KCMC are assigned a unique identification number which was retrieved and re-assigned for future deliveries to keep track of, and compile the medical record. These identification numbers were collected by the registry form at each birth and were used to link subsequent births of the same woman in the registry.

In order to ensure that births with the same maternal id-number were likely to be births of the same woman, we required that the linkage had to satisfy two criteria: 1). We matched the year of birth of the linked data with calendar years of previous births recorded in a reproductive history section of the questionnaire based on maternal recall and interview after each birth. We required that the reproductive history at the second birth included the birth year of the first (with an error margin of two years). 2) The birth interval calculated from birth dates of two linked births was cross-checked against the calculated change in maternal age. Linked births with a discrepancy of more than 2 years were excluded from the data.

### Description of the cohort

**Figure 1:**
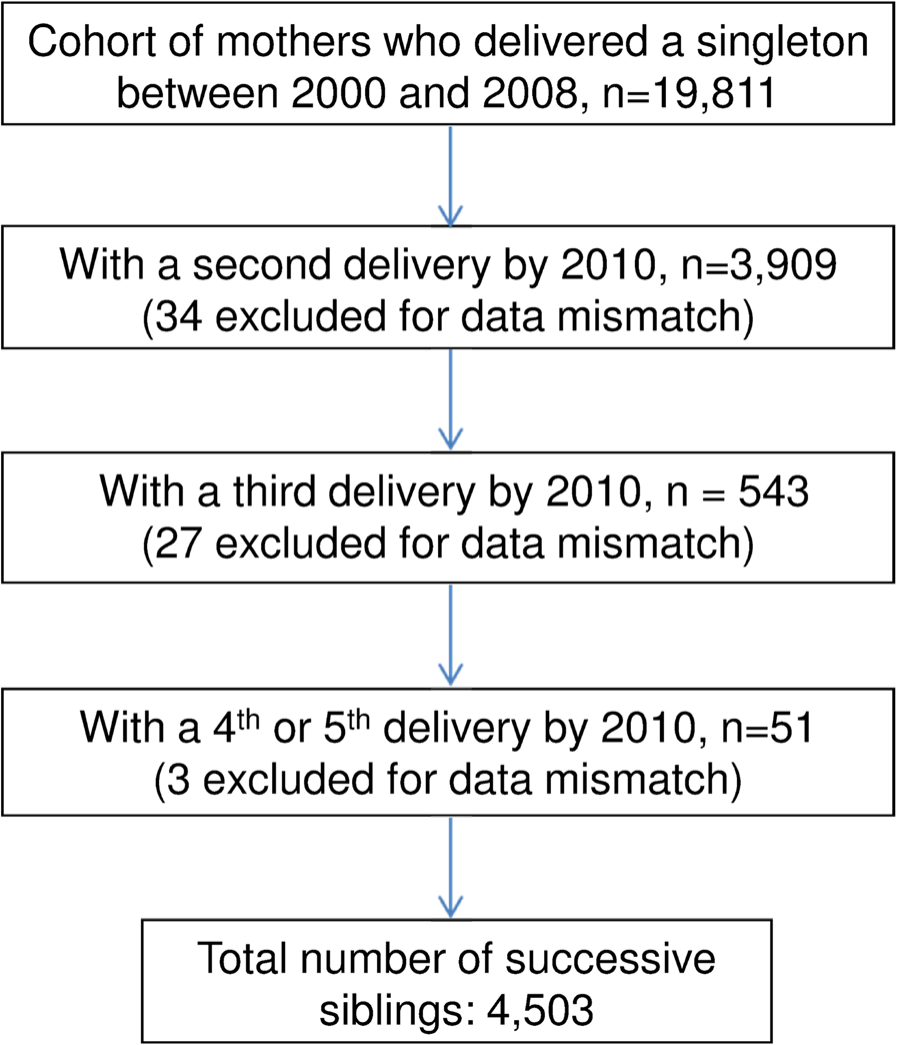
Schematic diagram of cohort follow-up of singleton deliveries.

There were 22,536 women who were recorded to have had deliveries at KCMC between 2000 and 2008. We restricted our study to women who had singleton delivery. After the exclusion of the women who were referred to KCMC from distant areas for various medical reasons or who had a multiple pregnancy, a total of 19,811 women who were recorded for the first time with a singleton delivery between year 2000 and 2008 were identified as our cohort (Figure 1). We then followed the cohort for new births in the whole period up to 2010 and identified all subsequent births recorded in the registry by record linkage. This allowed for a follow-up period of at least 2 years for all women and a median follow-up of 6.6 years. Women who experienced perinatal death in their first study pregnancy (n = 875) formed an exposed group, and those who had surviving babies (n = 18,936) formed an unexposed group within the cohort.

A total of 3,909 (19.7%) women in the cohort were recorded with at least one or more delivery within the follow-up period (Figure 1). Those 3,909 contributed a total of 4,503 subsequent births in the cohort. We measured recurrence from the first birth of women in the cohort to any of the subsequent births. The first pregnancy would here refer to the first pregnancy of a mother that was recorded in the birth registry and not necessarily her own first pregnancy.

### Main variables and statistical analysis

The main outcome of interest was perinatal death in a subsequent pregnancy. Perinatal mortality was defined as the sum of stillbirths and early neonatal deaths. Stillbirth was defined as a fetal death that occurred at 28 weeks of gestation age (antepartum death) or during labour (intrapartum death) or in a baby of at least 500 grams. Early neonatal death was defined as the death of a live-born infant during the first 7 days of life. Independent variables include maternal and fetal characteristics in the first recorded pregnancy that were used to predict risk in a subsequent pregnancy (Tables 1 and 2).

**Table 1:** Socio-demographic characteristics of the 3,909 women in the cohort at first birth

		**Outcome in the 1st pregnancy**		
**Maternal characteristics (1st pregnancy)**	**Total**	**No-perinatal death**	**Perinatal-death (%)**	P- value*
Education level				0.19
≤ 12 years	2,590	2,400	190 (7.3)	
>12 years	1,319	1,237	82 (6.2)	
Body Mass Index**				0.06
Underweight (<18.5)	520	463	57 (11.0)	
Normal (18.5-24.9)	204	183	21 (10.3)	
Overweight (25–29.9)	302	285	17 (5.6)	
Obese (>= 30)	216	201	15 (7.7)	
Number of ANC visits				<0.001
< 5	2,231	2,028	203 (8.8)	
>= 5	1,678	1,609	69 (4.3)	
Maternal age: mean (SD^†^)		25.9 (4.9)	26.3 (5.2)	0.94
All women	3,909	3,637	272 (6.9)	

*Chi-square tests for heterogeneity except of a t-test for mean maternal age.

†SD – Standard deviation.

**The number do not add to the total due to missing BMI variables (weight or height).

**Table 2:** Clinical subgroups at first birth of the 3,909 women in the cohort who had a second birth

	**Outcome in the 1st pregnancy**		
**Clinical subgroups (1st pregnancy)**	**Total**	**Perinatal death n (%)**	P- value*
**All women**	**3,909**	**272 (6.9)**	
Caesarian- section			
Yes	1,253	90 (7.2)	0.59
No	2,656	182 (6.8)	
Preeclampsia			
Yes	137	26 (18.9)	<0.001
No	3,772	246 (6.5)	
Abruption placenta			
Yes	23	14 (60.9)	<0.001
No	3,886	258 (6.6)	
Placenta Previa			
Yes	12	1 (8.3)	0.85
No	3,897	271 (6.9)	
Induced labour			
Yes	1,519	130 (8.6)	0.002
No	2,390	142 (5.9)	
Preterm Delivery (<37 weeks)^**†**^			
Yes	508	101 (19.9)	<0.001
No	3,098	150 (4.8)	
Low birth weight (<2500 g)			
Yes	575	129 (22.4)	<0.001
No	3,334	143 (4.2)	
Infections			
Yes	1,634	99 (6.1)	0.06
No	2,275	173 (7.6)	

*Chi-square test.

†Total do not add to 3,909 because 303(7.8%) mothers missed gestational age.

Statistical analysis was performed using IBM SPSS version 18.0 [12] and Stata version 11.0 [13]. Means were compared between groups using t-tests for continuous variables. Perinatal mortality was calculated as a proportion per 1000 births. The continuation rate was expressed as the proportion of women in the cohort who had a second recorded pregnancy within the follow-up period. Since absolute risks were higher than 5%, the relative risk of recurrence of perinatal death with 95% confidence intervals was estimated using log-binomial regression models with adjustment for potential confounders. Log-binomial models were also used to test for difference between recurrence risks by introducing interaction terms in the models. Mothers were the primary unit of analysis. Since we included recurrence from the first to any of the subsequently recorded births of the same mother, our analysis involved correlated data whenever a mother contributed more than one follow-up birth. We used a clustered analysis technique with robust estimation of variances to account for repeated observations from the same mother. By using retrospective information in the registry regarding a mother’s reproductive history, we were also able to calculate the rates of women with prior stillbirths to compare with recurrence risks. The study was approved by the Kilimanjaro Christian Medical College research ethics committee.

## Results

Of the 3,909 women who delivered singletons for the subsequent pregnancy and contributed in the follow-up of the cohort, 272 (6.9%) lost their child in a perinatal death in their first recorded pregnancy. Women who lost their child in their first recorded pregnancy were more likely to have had preeclampsia, abruption placenta, induced labour, preterm delivery, maternal underweight, attendance to antenatal care (<5 visits) and low birth weight babies as compared with women who has a surviving child (Tables 1 and 2).

### Risk of perinatal death in a subsequent pregnancy

Women who lost their child in the first recorded pregnancy were more likely than women with a surviving child to be recorded with a subsequent pregnancy (31% versus 19%). Table 3 shows that, the recurrence risk of perinatal death for women who lost their child in the first recorded pregnancy was 90.6 per 1000 births compared with a risk of 27.6 per 1000 births for those who had a surviving baby from a previous pregnancy. This amount to a 3.2-fold relative risk (95% CI: 2.2 - 4.7) for mothers who lost their child in first reported pregnancy. Recurrence constituted 21.2% (31/146) of perinatal deaths in subsequent births. We found no difference in recurrence risk for the pregnancy following immediately after a perinatal death and later pregnancies (data not shown).

**Table 3:** Predictors of perinatal death in next pregnancy by maternal conditions in the first pregnancy

		**Perinatal death in a subsequent pregnancy**		
**Maternal conditions**	**Babies at risk**	**n (%)**	RR* (95% CI^**†**^**)**^**‡**^	**P-value**^**‡**^
Perinatal mortality				
Yes	342	31 (9.1)	3.2 (2.2 - 4.7)	<0.001
No	4,161	115 (2.8)	Reference	
Preeclampsia				
Yes	149	22 (14.8)	4.5 (2.9 - 7.1)	<0.001
No	4,208	124 (2.9)	Reference	
Preterm delivery				
Yes	545	65 (11.9)	5.8 (4.1 - 8.0)	<0.001
No	3,476	66 (1.9)	Reference	
Low birth weight				
Yes	613	78 (12.7)	6.5 (4.7 - 8.9)	<0.001
No	3,724	66 (1.8)	Reference	
Caesarea section				
Yes	1,392	59 (4.2)	1.4 (1.0 - 1.9)	0.04
No	2,947	87 (2.9)	Reference	
Induced labour				
Yes	1,727	66 (3.8)	1.3 (0.9 - 1.8)	0.15
No	2,630	80 (3.0)	Reference	
Infections				
Yes	1,852	53 (2.9)	0.8 (0.6 - 1.1)	0.14
No	2,505	93 (3.7)	Reference	

*RR: adjusted relative risk.

†CI: confidence interval.

‡Adjusted for maternal age and education attainment in Log-binomial model accounting for correlation between successive deliveries of the same mother.

Some other conditions of the first pregnancy were also independent predictors of perinatal mortality in the subsequent pregnancy for all women; these include preeclampsia (RR = 4.5; 95% CI: 2.9 - 7.1), preterm delivery (RR = 5.8; 95% CI: 4.1 - 8.0) and low birth weight (RR = 6.5; 4.7 - 8.9). These factors also had an influence on the recurrence risk of perinatal death (Table 4). The highest recurrence risk of perinatal death was observed when the previous pregnancy was complicated by preeclampsia (7/35, 20%). Mothers who lost a baby in the previous pregnancy who was born preterm or low birth weight had a 14% risk of having a perinatal death in a consecutive pregnancy. The risk of perinatal death in a consecutive pregnancy was still high (10%) even for mothers whose previous baby survived. The lowest perinatal mortality in a subsequent birth was observed when the previous baby had normal birth weight (not LBW) and was not recorded as a perinatal death (59/3,620, 1.6%).

**Table 4:** Continuation rate and recurrence risk of perinatal death by characteristics at the first pregnancy

				**Perinatal death in a subsequent pregnancy**				
**Characteristic at first birth**	**Survival of first baby**	Continuation rate*	**Number at risk**	**Perinatal deaths (n)**	**Risk (%)**	**RR**^**†**^	**95% CI**^**‡**^	**p difference of RRs**
Preeclampsia								
Yes	Per. death	0.27	35	7	20	1.8	0.8 – 3.9	0.20
	No per. death	0.17	136	15	11.0			
No	Per. death	0.32	307	24	7.8	3.1	2.1 – 4.7	
	No per. death	0.24	4,025	100	2.5			
LBW								
Yes	Per. death	0.32	160	22	13.8	1.3	0.8 – 1.9	0.02
	No per. death	0.25	531	56	10.5			
No	Per. death	0.31	182	9	4.9	3.2	1.6 – 6.1	
	No per. death	0.19	3,620	59	1.6			
Preterm birth								
Yes	Per. death	0.32	126	17	13.5	1.3	0.8- 2.1	0.01
	No per. death	0.23	484	48	9.9			
No	Per. death	0.33	216	14	6.5	3.9	2.2 - 7.0	
	No per. death	0.19	3,677	67	1.8			
C/section								
Yes	Per. death	0.33	120	11	9.2	2.5	1.4 – 4.6	0.30
	No per. death	0.20	1,331	48	3.6			
No	Per. death	0.31	222	20	9.0	3.8	2.4 – 6.1	
	No per. death	0.19	2,830	67	2.4			
Induced labour								
Yes	Per. death	0.37	165	12	10.3	2.2	1.2 – 3.9	0.07
	No per. death	0.25	1,628	54	3.3			
No	Per. death	0.27	177	19	10.7	4.4	2.7– 7.1	
	No per. death	0.17	2,533	61	2.4			
ANC visits								
>= 5	Per. death	0.41	90	5	5.6	3.4	2.4 – 4.9	0.25
	No per. death	0.23	1,784	45	2.5			
<5	Per. death	0.29	252	26	10.3	6.7	1.9 – 7.6	
	No per. death	0.17	2,377	70	2.9			
Infections								
Yes	Per. death	0.32	129	13	10.1	4.5	2.6 – 7.7	0.17
	No per. death	0.20	1,776	40	2.6			
No	Per. death	0.31	213	18	8.5	2.7	1.6 – 4.4	
	No per. death	0.18	2,385	75	3.1			

*Proportion of women with a second birth.

†RR: adjusted relative risk adjusted for maternal age and education attainment.

‡CI: confidence interval from log-binomial models accounting for correlation between successive deliveries of the same mother.

In effect, the relative risk of recurrence of perinatal death was high when the previous baby had a normal birth weight (3.2; 95% CI: 1.6-6.1), but low when the previous baby had low birth weight (1.3; 95% CI: 0.8-1.9) since all women with a previous low birth weight baby had high risks (interaction p-difference = 0.02). Similarly, the relative risk of recurrence of perinatal death was high when the previous baby was born at term (3.9; 95% CI: 2.2-7.0), but was low when the previous baby was born preterm (1.3; 95% CI: 0.8-2.1) (p-difference = 0.01). Recurrence risks of perinatal death were not significantly affected by conditions like C-section, induction of delivery, infection or the number of antenatal care visits in the previous pregnancy (Table 4).

### Risk of stillbirth or early neonatal death

We performed a sub-study of stillbirth separate from early neonatal death. There was a tendency for mothers to re-experience a death in the same sub-category of perinatal death as in her first pregnancy. If a woman had lost her first baby due to stillbirth, she had a 5.1-fold (95% CI: 3.2 - 8.1) risk of losing her next baby due to stillbirth, while her risk of early neonatal death in the next pregnancy was 2.2-fold (95% CI: 1.0-4.9) (Table 5).

**Table 5:** Recurrence risk of stillbirth and early neonatal death in a subsequent pregnancy

		**Recurrence risk in a subsequent pregnancy**			
		**Stillbirth**		**Neonatal death**	
**First pregnancy**	**At risk**	**n (%)**	**RR (95% CI)**	**n (%)**	**RR (95% CI)**
Stillbirth					
Yes	253	23 (9.1)	5.1 (3.2 - 8.1)	9 (3.6)	2.2 (1.0 - 4.9)
No	4,250	76 (1.8)	reference	72 (1.7)	reference
Neonatal death					
Yes	139	2 (1.4)	0.5 (0.1 - 3.5)	5 (3.6)	2.1 (0.5 - 7.9)
No	4,265	97 (2.3)	reference	76 (1.8)	reference

## Discussion

From our data from Northern Tanzania we have previously described the perinatal mortality and the distribution of causes of perinatal death [6]. In the current paper we describe how perinatal death in one pregnancy is associated with perinatal death in the subsequent pregnancies. The perinatal death in one pregnancy was a strong predictor of the perinatal death in a subsequent pregnancy. Women who experienced perinatal death in a previous pregnancy had 9% increased risk of losing a baby in a subsequent pregnancy. The absolute recurrence risk of perinatal death was also high for mothers whose previous baby was born preterm or had low birth weight who died perinatally (13% to 14%). The lowest perinatal mortality in a subsequent birth (1.6%) was observed for women who had a previous surviving baby born with normal birth weight. These results suggest that there is great underlying heterogeneity in women’s risk of losing their offspring in this high risk area of sub Saharan Africa. We found no previous cohort studies from sub Saharan Africa that had reported prospectively estimated recurrence risks of pregnancy outcomes.

### Comparison with other studies

Although absolute risks were much higher than in western countries, the strength of association of a 3.2-fold recurrence risk observed in our data was consistent with associations observed in western countries [14-16]. Some studies have found slightly higher relative risks [9,17,18], and one study in Afro-American women estimated a four-fold recurrence risk [14]. A 2-fold recurrence risk has been reported from Israel [11]. All these studies reported absolute recurrence risks that were much lower than the recurrence risk of nine per cent estimated in this study. In western countries, increased antepartum surveillance may be undertaken in subsequent pregnancies for women with history of a perinatal loss. Health care providers may consider women with a previous loss as a high risk group, and therefore refer them for closer follow-up in their subsequent pregnancies. This may not be the case in the low-resourced settings where specialized care services are not available. It is surprising that the recurrence risk still appears to be around 3-fold.

Conditions in one pregnancy such as preeclampsia; preterm birth and low birth weight were also associated with increased risk of perinatal mortality in a subsequent pregnancy. This is in agreement with previous studies [19-21]. When any of these conditions occurred in one pregnancy, the risk was high for a perinatal death in the subsequent pregnancy regardless whether the baby had died or not in the previous pregnancy. These factors may share recurring underlying pathophysiological mechanisms that may also be associated with perinatal death.

When we separated stillbirths from early neonatal deaths, we found that women who experienced stillbirth in one pregnancy had a 5.1-fold increased risk of stillbirth in the next pregnancy. This is similar to the previously reported relative risk of stillbirth recurrence among relatively low-risk women in a Missouri maternally linked cohort (age <35 years; gestational age of 20–44 weeks; absence of congenital anomalies; non-smokers and singleton births)[18], but higher than estimates from population based studies from western countries [22-24]. The lower recurrence risk in the latter studies may be explained by the targeted antepartum surveillance by health professionals in the future pregnancies for women with a previous stillbirth.

In addition, the recurrence risk of early neonatal death in our study was 2.1-fold. This is similar to an estimated 2.8-fold risk in a recent report using survey data on maternal and perinatal health data from developing countries [25].

Using reproductive history data from interviews with the women, we were also able to estimate the risk of stillbirth in a previous pregnancy for women who had a stillbirth in the delivery recorded by the registry. This risk should be comparable with the recurrence risk, but slightly lower compared to the risk calculated from the prospective data (3.4-fold vs. 5.1-fold). Our study probably had under-ascertainment of perinatal deaths. Although stillbirths with birth weights as low as 600 grams were included in the study, we still may have an underrepresentation of early stillbirths.

### Limitations and strengths

Our study also had several strengths. The data contained detailed information on the pregnancy and delivery as well as the condition of the baby and the mother’s reproductive history. All data were collected by a standardized protocol by specially trained midwifes. Each delivering woman was identified by a unique id-number, which allowed linkage of subsequent births. This provided a unique opportunity to study recurrent pregnancy outcomes in a prospective study design.

The large sample size allowed us to estimate significant differences in the risk of perinatal death in subsequent deliveries depending on the outcome of a previous pregnancy. This opens the possibility that high-risk pregnant women in Tanzania or similar African settings may be identified and benefit from special clinical attention during their future pregnancies.

Our prospective study has several limitations. First, there is a possibility of loss to follow-up. We observed that 19.7% of the women in the cohort were observed with a subsequent birth within the follow-up time. The availability of reproductive history data from the interviews allowed us to estimate the proportion of women who should have two subsequent births within the follow-up time. A total of 21,086 women delivered a singleton between 2002 and 2010 in the registry. For women who had more than one birth, we identified and used data only from the most recent. A total of 7,191 (34.1%) of these women reported to have had a previous delivery in the period 2000 to 2010. Under an assumption of symmetric reproductive patterns in the period, this should be an estimate of the number of women in our cohort who should have had a subsequent birth within our follow-up time including births outside the hospital. Although this figure might be subject to incomplete recall, it was still higher than the 19.7% who were recorded with a subsequent birth in the prospective follow-up in the registry. Since 14.4% of the 34.1% who might have had subsequent births were lost in our follow-up, we may have lost 42% (14.4/34.1) of the women who had subsequent births in our follow-up of the cohort of 19 811 women.

With all the limitations of this comparison, it suggests that as many as 40% deliveries of the women in our cohort were lost in the follow-up either because they delivered elsewhere or because we failed to trace them in the hospital. If women who had delivered previously at the hospital were assigned a new id-number in their next delivery, our mechanism of follow-up would fail. There is a possibility that some women with perinatal loss in their first pregnancy are more likely to change the hospital for the next delivery because they may associate the perinatal loss to substandard care in the previous facility. As well they may not want to be reminded of the previous loss; if this happened in some occasions, these women might have been assigned with a new id-number. We had no opportunity to identify such women or to estimate the magnitude of this problem. Such loss to follow-up may lead to bias in the recurrence risks (assuming that assignment of a new hospital number is dependent of the outcome of the previous pregnancy).

Second, there is a possibility of selection bias since women who experience perinatal loss or pregnancy complications in their first pregnancy may tend to deliver more often at this hospital in their subsequent pregnancy compared to women who deliver a healthy child or had normal pregnancy. If women who come back to the hospital for a future delivery after a previous loss have a risk of losing their next baby which is different from the risk of women who do not come back after a previous loss, our estimates of recurrence risk could be biased due to overrepresentation of the high risk women. However, in a sub analysis, we excluded all women in the cohort who were referred for medical reasons for their subsequent delivery at KCMC. The results were very similar with a slightly higher relative risk of recurrence of perinatal death (relative risk of 3.5 vs 3.2 in the main analysis). There was no indication of bias caused by overrepresentation in our follow up of women with a previous loss who had particular problems in their second pregnancy.

Women who experienced perinatal death in their first study pregnancy were more likely to be observed with a subsequent pregnancy compared to those who had surviving children (32% versus 19%). Similar findings have been reported elsewhere [15,24]. For our data this may be partly explained by selective fertility, which is defined as the tendency for a woman to replace a perinatal loss with a new pregnancy until the desired number of children is attained. Selective fertility may depend both on the survival rate of babies in the population and the general fertility, and we do not know the magnitude of selective fertility in Tanzania. For our data, it is possible that women with a previous loss were more likely to show up at KCMC for a future delivery. This would bias estimation of selective fertility.

Third, there was a possibility of underestimation of recurrent risk of early neonatal death as well as of perinatal death. Our follow-up was limited to stillbirths and early neonatal death that occurred in the hospital. The early neonatal deaths that occurred outside the hospital after a mother with uncomplicated births and her baby were discharged were not identified. We may have had under ascertainment of neonatal deaths that occurred outside the hospital.

## Conclusions

The risk of recurrence of perinatal death for women in Northern Tanzania was 9.1% compared to a much lower risk for women with a surviving child from a previous pregnancy. Pregnant women in Tanzania with a previous perinatal loss, preterm birth or low-birth weight infant may require special clinical attention even in a resource limited setting. High risk of recurrence of perinatal death also among women with previous term or normal birth weight babies may suggest problems in delivery management. Health care providers should consider deliveries of all mothers with a previous perinatal loss as high-risk delivery. Prenatal and neonatal surveillance should be considered in future births and clinical counseling regarding future risk should be provided. Further community or population-based studies are needed to confirm the estimated risks and to identify contributing factors. Intervention studies are needed to study effects of measures taken to reduce the risk of recurrence of perinatal death.

## Competing interests

The authors declared that they have no competing interests.

## Authors’ contributions

MJM: conception idea for the study, design of the study, manuscript preparation and subsequent revisions, RTL: conception of the research idea, advice in designing the study and revision of the manuscript for important intellectual content. RM, JO, AKD and BTM: reviewed the manuscript, GM is responsible for the KCMC registry. All authors read and approved the final manuscript.

## Pre-publication history

The pre-publication history for this paper can be accessed here:

http://www.biomedcentral.com/1471-2393/13/166/prepub
